# Enantioselective total synthesis of (*R*)-(−)-complanine

**DOI:** 10.3762/bjoc.8.192

**Published:** 2012-10-04

**Authors:** Krystal A D Kamanos, Jonathan M Withey

**Affiliations:** 1 Department of Physical Sciences, Grant MacEwan University, 10700 104 Ave, Edmonton, T5J 4S2, Canada

**Keywords:** complanine, enantioselective synthesis, marine fireworm, nitrosoaldol, organocatalysis

## Abstract

A route is described for the enantioselective synthesis of (*R*)-(−)-complanine, a marine natural product isolated from *Eurythoe complanata*, and known to be a causative agent in inflammation. An organocatalytic, asymmetric oxyamination of a homoconjugated all-*Z*-dienal intermediate provides versatile and efficient access to the natural product.

## Introduction

The marine fireworm *Eurythoe complanata* resides in the shallow water and sands of temperate and sub-tropical regions. Its small setae cause skin inflammation upon contact, the causative agent having been identified as complanine. This novel amphipathic substance was first isolated from *Eurythoe complanata* in 2008, by Nakamura and Uemura [1]. Complanine induces inflammation by activating PKC (protein kinase C) in the presence of Ca^2+^ and TPA (12-*O*-tetradecanoylphorbol 13-acetate). PKC plays an important role in inflammation, namely through control of signal transduction cascades, and the biological activity of complanine may be understood in terms of controlling these cascades.

**Figure 1:**
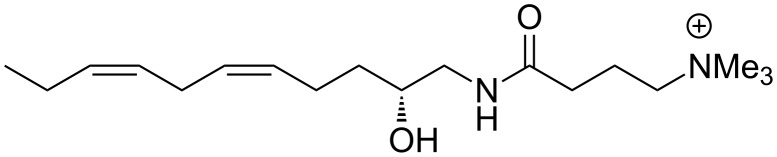
Structure of (*R*)-(−)-complanine.

From a structural perspective, complanine contains a novel trimethylammonium cationic group and can be characterized as possessing a homoconjugated all-*Z*-diene moiety and an amino alcohol *N*-acylated with a γ-aminobutyric acid (GABA) derivative. Complanine is structurally related to the obscuraminols, isolated from another marine source, the ascidian *Pseudodistoma obscurum* [2]. Unlike complanine, however, these substances are simple amino alcohols that do not possess any other chemical functionalities. Synthetic studies, also conducted by Nakamura and Uemura, established the absolute structure and configuration as (*R*)-(−)-complanine (Figure 1), showing it to be related to other natural products that possess the vicinal amino alcohol moiety. Using a chiral-synthon approach, (*R*)-(−)-complanine was synthesized in a sequence of nine linear steps, beginning with (*R*)-malic acid [3].

As part of our research endeavours directed towards the organocatalytic synthesis of 1,2-amino alcohols, we have developed a new asymmetric strategy leading to (*R*)-(−)-complanine. This approach utilizes an asymmetric organocatalytic *O*-nitrosoaldol reaction as the source of chirality, with accompanying amination of the aldehyde functional group resulting in a concise and efficient synthesis.

## Results and Discussion

**Scheme 1:**
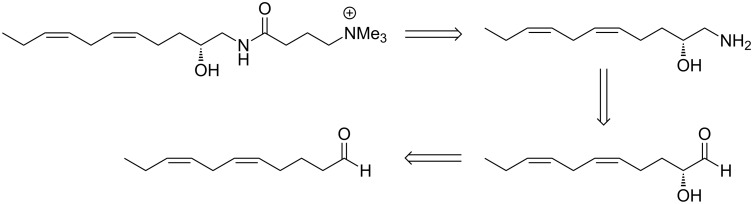
Retrosynthetic analysis of (*R*)-(−)-complanine.

Herein we report the concise and efficient synthesis of (*R*)-(−)-complanine, via a highly effective organocatalytic *O*-nitrosoaldol as the key step. A retrosynthetic analysis for complanine is depicted in Scheme 1.

**Scheme 2:**
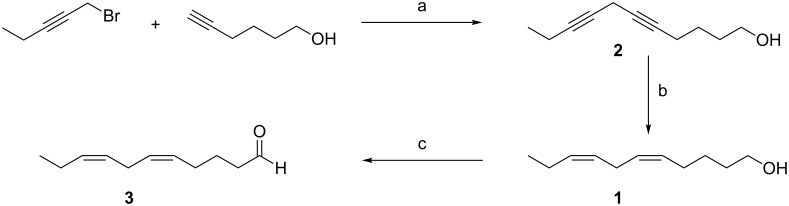
Reagents and conditions: (a) Cs_2_CO_3_, CuI, TBAI, DMF, rt, 24 h, 91%; (b) H_2_ (1 atm), Lindlar catalyst, EtOAc, pyridine, rt, 24 h; (c) DMSO, (COCl)_2_, DCM, −78 °C, then; **1**, −78 °C, 45 min, then, Et_3_N, 0 °C, 1.5 h, 86% from **2**.

The synthesis was initiated by preparation of homoconjugated all-*Z*-diene **1** (Scheme 2). Sequential olefin preparation by using Wittig chemistry, or selective polyyne reduction represent two traditional methods for the preparation of homoconjugated all-*Z*-polyenes. In the latter, metal-mediated coupling of 1-alkynes and propargylic halides is generally employed, with copper (I) alkynides being the least prone to isomerization, polymerization or multiple alkylation. Of the several methods reported, a modification of the cesium carbonate-promoted coupling developed by Caruso and Spinella provided the most efficient conditions [4]. Thus, commercially available and unprotected 5-hexyn-1-ol underwent smooth coupling with 1-bromopent-2-yne in *N*,*N*-dimethylformamide, in the presence of stoichiometric amounts of cesium carbonate, copper iodide and tetrabutylammonium iodide. The resulting skipped diyne **2** was selectively reduced to homoconjugated all-*Z*-diene **1**, with subsequent Swern oxidation affording aldehyde **3**, in 86% yield from **2**, as a key intermediate in the total synthesis.

**Scheme 3:**
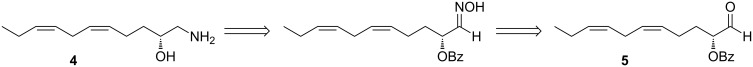
Direct approach to amino alcohol **4**.

With **3** in hand, attention was then turned to its conversion to amino alcohol **4**. It was envisioned that such conversion could be effected in a one-pot reaction by employing lithium aluminium hydride reduction of an aldoximine [5], formed in situ by condensation of a suitably oxygenated aldehyde **5** with hydroxylamine hydrochloride (Scheme 3).

Although a number of acyloxylations have been reported, the direct catalytic asymmetric acyloxylation of aldehydes has only been recently realized [6–7]. Thus, the asymmetric benzoylation of aldehydes, according to the method of Tomkinson [7], was attempted. Utilizing benzoyl peroxide in the presence of MacMillan imidazolidinone (5*R*)-2,2,3-trimethyl-5-benzyl-4-imidazolidinone [8] with 4-nitrobenzoic acid as cocatalyst, all efforts to effect benzoylation of **3**, yielding **5**, were unsuccessful, and this route was ultimately abandoned as attention turned to more conventional α-oxygenation strategies and a stepwise approach to **4**.

**Scheme 4:**
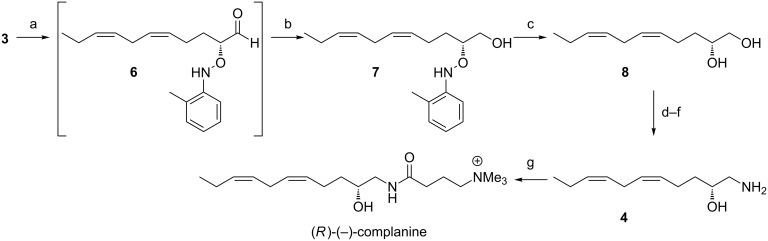
Reagents and conditions: (a) 2-Nitrosotoluene, L-proline (10 mol %), CHCl_3_, 0 °C, 3 h; (b) NaBH_4_, EtOH, 0 °C, 30 min, 78% from **3**; (c) Cu(OAc)_2_, EtOH, rt, 16 h, 72%; (d) MsCl, pyridine, CH_2_Cl_2_, 0 °C, 3 h; (e) NaN_3_, DMF, 80 °C, 16 h, 84% from **8**; (f) PPh_3_, THF, H_2_O, rt, 12 h, 88%; (g) *N*-[4-(trimethylammonio)butyryloxy]succinimide iodide, MeOH, rt, 16 h, 62%.

The reaction of aldehydes with nitrosobenzene in the presence of proline-derived secondary amine catalysts represents the current benchmark in organocatalytic α-oxygenation strategies [9]. A known drawback of this approach is the instability of the oxyaminated products, presumably owing to N–O bond lability. Recent reports suggest that this *O*-nitrosoaldol reaction proceeds much more cleanly with 2-nitrosotoluene than with nitrosobenzene, probably owing to the suppression of N–O bond cleavage [10]. Thus, treatment of aldehyde **3** with 2-nitrosotoluene in the presence of L-proline as catalyst, resulted in clean α-oxygenation (Scheme 4). Attempts to effect the direct conversion of **7** to amino alcohol **4**, by using the aldoximine approach described above (Scheme 3), yielded a complex mixture of products. However, **6** was readily isolated as oxyaminated alcohol **7**, following in situ reduction with sodium borohydride. Subsequent copper-mediated N–O bond cleavage gave chiral diol **8** in 97% ee (determined by HPLC analysis using a chiral stationary phase). Diol **8** had identical spectroscopic properties compared to those reported by Nakamura and Uemura and intercepted their synthesis of (*R*)-(−)-complanine [3]. Thus, the conversion of **8** to the corresponding amino alcohol was readily effected by azide formation and subsequent reduction. In an analogous fashion, the activated ester of 4-(trimethylammonio)butanoate (synthesized from γ-aminobutyric acid [11]) underwent reaction with amino alcohol **4** to furnish (*R*)-(−)-complanine in 62% yield.

The isolated, synthetic complanine was identical in all its spectral data to the previously reported natural material [3], with an optical rotation in reasonable agreement ([α]_D_^22^ −12.1 (*c* 0.85, H_2_O), lit. −9.9 (*c* 0.12, H_2_O)).

## Conclusion

An efficient and enantioselective route to (*R*)-(−)-complanine was developed, by using an organocatalytic α-oxygenation strategy. This flexible approach also permits ready access to the (*S*)-enantiomer of the natural product and offers an alternate route to the synthesis of recently isolated neocomplanines A and B [12], along with the obscuraminols [2]. Such synthetic studies are currently underway in our laboratory.

## Experimental

**Undeca-5,8-diyn-1-ol (2):** To a suspension of anyhydrous Cs_2_CO_3_ (9.19 g, 28.2 mmol), TBAI (10.39 g, 28.2 mmol) and CuI (5.37 g, 28.2 mmol) in anhydrous DMF (60 mL) was added 5-hexyn-1-ol (3.11 mL, 28.2 mmol). Following stirring at rt for 20 min, 1-bromopent-2-yne (2.88 mL, 28.2 mmol) was added dropwise. The reaction mixture was stirred for an additional 24 h at rt before addition of NH_4_Cl (aq, sat, 100 mL) and extraction of the aqueous phase with Et_2_O (3 × 100 mL). The organic layer was dried (MgSO_4_), filtered and concentrated in vacuo. Purification by flash column chromatography (silica gel, hexane/ethyl acetate, 9:1) gave undeca-5,8-diyn-1-ol (**2**) as a colorless oil (4.21 g, 91%): IR ν_max_ (thin film)/cm^−1^ 3328, 3019, 2941, 2244, 1447, 1048, 976, 788, 698; ^1^H NMR (400 MHz, CDCl_3_) δ 1.12 (3H, m, C11H_3_), 1.51–1.68 (4H, m, C2H_2_ and C3H_2_), 2.20 (2H, m, C10H_2_), 3.12 (2H, m, C7H_2_), 3.63 (2H, m, C1H_2_); ^13^C NMR (100 MHz, CDCl_3_) δ 5.6 (C7), 9.6 (C10), 12.4 (C11), 18.5 (C4), 25.0 and 32.0 (C2 and C3), 62.0 (C1), 74.4, 75.7, 79.2 and 82.1 (C5, C6, C8 and C9); HRMS–ESI [M + H]^+^ calcd for C_11_H_17_O, 165.1279; found, 165.1282.

**(5*Z*,8*Z*)-Undeca-5,8-dien-1-ol (1):** To a solution of undeca-5,8-diyn-1-ol (**2**, 4.01 g, 24.4 mmol) in ethyl acetate/pyridine (9:1, 50 mL) was added Lindlar’s catalyst (1.51 g). The mixture was stirred at rt under a hydrogen atmosphere for 24 h. The reaction mixture was filtered through a pad of celite and concentrated in vacuo to yield (5*Z*,8*Z*)-undeca-5,8-dien-1-ol (**1**, 4.26 g) of sufficient purity to be used directly in the next step. Purification of an analytical sample by flash column chromatography (silica gel, hexane/ethyl acetate, 9:1) gave (5*Z*,8*Z*)-undeca-5,8-dien-1-ol (**1**) as a colorless oil: IR ν_max_ (thin film)/cm^−1^ 3281, 3022, 2934, 2865, 1646, 1457, 1052, 1021, 928, 768, 694; ^1^H NMR (400 MHz, CDCl_3_) δ 0.97 (3H, t, *J =* 7.6 Hz, C11H_3_), 1.44 (2H, m, C3H_2_), 1.58 (2H, m, C2H_2_), 2.09 (4H, m, C2H_2_ and C3H_2_), 2.78 (2H, m, C7H_2_), 3.65 (2H, t, *J =* 6.9 Hz, C1H_2_), 5.28–5.44 (4H, m, C5H, C6H, C8H and C9H); ^13^C NMR (100 MHz, CDCl_3_) δ 14.3 (C11), 20.5 (C10), 25.6 and 25.8 (C3 and C7), 26.9 (C4), 32.4 (C2), 62.9 (C1), 127.3, 128.5, 129.6 and 131.9 (C5, C6, C8 and C9); HRMS–ESI [M + H]^+^ calcd for C_11_H_21_O, 169.1592; found, 169.1593.

**(5*Z*,8*Z*)-Undeca-5,8-dienal (3):** A solution of DMSO (4.76 mL, 67.0 mmol) in CH_2_Cl_2_ (50 mL) was added dropwise to a solution of oxalyl chloride (2.83 mL, 33.5 mmol) in CH_2_Cl_2_ at −78 °C. Following stirring for 20 min, a solution of (5*Z*,8*Z*)-undeca-5,8-dien-1-ol (**1**, 3.76 g, 22.3 mmol) in CH_2_Cl_2_ (50 mL) was added dropwise to the reaction mixture. After stirring for an additional 45 min at −78 °C, Et_3_N (18.7 mL, 134 mmol) was added dropwise, and the reaction mixture was warmed to 0 °C, whereat it was stirred for 1.5 h. Water (100 mL) was added and the reaction mixture was warmed to rt. The aqueous phase was extracted with CH_2_Cl_2_ (3 × 50 mL) and the combined organic layers were dried (MgSO_4_), filtered and concentrated in vacuo. Purification by flash column chromatography (silica gel, hexane/ethyl acetate, 20:1) gave (5*Z*,8*Z*)-undeca-5,8-dienal (**3**) as a colorless oil (3.08 g, 86% from **2**): IR ν_max_ (thin film)/cm^−1^ 3036, 2940, 2860, 2748, 1719, 1644, 1455, 1207, 921, 875, 841, 698; ^1^H NMR (400 MHz, CDCl_3_) δ 0.97 (3H, t, *J =* 7.7 Hz, C11H_3_), 1.71 (2H, quintet, *J* = 7.7 Hz, C3H_2_), 2.01–2.17 (4H, m, C4H_2_ and C10H_2_), 2.44 (2H, td, *J* = 7.9 Hz, 2.1, C2H_2_), 2.77 (2H, t, *J* = 5.6 Hz, C7H_2_), 5.24–5.45 (4H, m, C5H, C6H, C8H and C9H), 9.77 (1H, t, *J* = 2.1 Hz, CHO); ^13^C NMR (100 MHz, CDCl_3_) δ 14.1 (C11), 20.6 (C10), 22.0 (C3), 25.6 (C7), 26.5 (C4), 43.3 (C2), 124.1, 124.9, 129.6 and 132.1 (C5, C6, C8 and C9), 201.2 (CHO); HRMS–ESI [M + H]^+^ calcd for C_11_H_19_O, 167.1436; found, 167.1432.

**(2*R*,5*Z*,8*Z*)-2-(*N*-*o*-Tolylaminooxy)-undeca-5,8-dien-1-ol (7):** To a solution of 2-nitrosotoluene (403 mg, 3.33 mmol) and L-proline (115 mg, 1.00 mmol) in CHCl_3_ (20 mL) at 0 °C was added a solution of (5*Z*,8*Z*)-undeca-5,8-dienal (**3**, 1.66 g, 10.0 mmol) in CHCl_3_ (20 mL). Following stirring at 0 °C for 3 h, a solution of NaBH_4_ (570 mg, 15.0 mmol) in EtOH (30 mL) was added and the reaction mixture was stirred for an additional 30 min at 0 °C. NaHCO_3_ (aq, sat, 100 mL) was added and the reaction mixture was warmed to rt. The aqueous phase was extracted with CHCl_3_ (3 × 50 mL) and the combined organic layers were dried (MgSO_4_), filtered and concentrated in vacuo. Purification by flash column chromatography (silica gel, hexane/ethyl acetate, 10:1→4:1) gave (2*R*,5*Z*,8*Z*)-2-(*N*-*o*-tolylaminooxy)-undeca-5,8-dien-1-ol (**7**) as a colorless oil (752 mg, 78%): [α]_D_^22^ −37.1 (*c* 1.00, CHCl_3_); IR ν_max_ (thin film)/cm^−1^ 3280, 3031, 2941, 2865, 1641, 1606, 1586, 1487, 1449, 1308, 1247, 1133, 1097, 1072, 1032, 1008, 926, 888, 839, 798, 748, 705; ^1^H NMR (400 MHz, CDCl_3_) δ 0.96 (3H, t, *J =* 7.8 Hz, C11H_3_), 1.56 (2H, m, C3H_2_), 2.04 (2H, m, C4H_2_), 2.12 (3H, s, ArCH_3_), 2.21 (2H, m, C10H_2_), 2.79 (2H, m, C7H_2_), 3.76 (1H, dd, *J* = 14.2, 8.6 Hz, C1H_A_), 3.85 (1H, dd, *J* = 14.2, 4.1 Hz, C1H_B_), 3.96 (1H, m, C2H), 5.26–5.48 (4H, m, C5H, C6H, C8H and C9H), 6.89 (1H, t, *J* = 7.3 Hz, ArH), 7.05 (1H, d, *J* = 7.4 Hz, ArH), 7.18 (1H, t, *J* = 7.1 Hz, ArH), 7.23 (1H, d, *J* = 7.3 Hz, ArH); ^13^C NMR (100 MHz, CDCl_3_) δ 14.2 (C11), 17.6 (ArCH_3_), 20.1 (C10), 22.3 (C4), 24.8 (C7), 31.6 C3, 66.2 (C1), 78.8 (C2), 114.6, 122.0, 123.6, 126.8, 130.3 and 145.9 (Ar); HRMS–ESI [M + H]^+^ calcd for C_18_H_28_NO_2_, 290.2120; found, 290.2122.

**(2*R*,5*Z*,8*Z*)-Undeca-5,8-diene-1,2-diol (8):** To a solution of (2*R*,5*Z*,8*Z*)-2-(*N*-*o*-tolylaminooxy)-undeca-5,8-dien-1-ol (**7**, 627 mg, 2.16 mmol) in EtOH (30 mL) was added Cu(OAc)_2_ (400 mg, 2.20 mmol) and the reaction mixture was stirred at rt for 16 h. NH_4_Cl (aq, sat, 50 mL) was added and the aqueous phase was extracted with EtOAc (3 × 30 mL). The combined organic layers were dried (MgSO_4_), filtered and concentrated in vacuo. Purification by flash column chromatography (silica gel, hexane/ethyl acetate, 4:1) gave (2*R*,5*Z*,8*Z*)-undeca-5,8-diene-1,2-diol (**8**) as a colorless oil (286 mg, 72%): [α]_D_^22^ −2.3 (*c* 0.90, CHCl_3_), lit. −2.0 (*c* 0.40, CHCl_3_); IR ν_max_ (thin film)/cm^−1^ 3256, 3035, 2938, 2871, 1639, 1486, 1449, 1107, 1052, 1032, 1008, 786, 738, 702; ^1^H NMR (400 MHz, CDCl_3_) δ 0.97 (3H, t, *J* = 7.7 Hz, C11H_3_), 1.50 (2H, m, C3H_2_), 2.07 (2H, m, C4H_2_), 2.20 (2H, m, C10H_2_), 2.80 (2H, t, *J* = 5.3 Hz, C7H_2_), 3.45 (1H, dd, *J* = 13.9, 8.1 Hz, C1H_A_), 3.63 (1H, dd, *J* = 13.9, 4.2 Hz, C1H_B_), 3.72 (1H, m, C2H), 5.27–5.44 (4H, m, C5H, C6H, C8H and C9H); ^13^C NMR (100 MHz, CDCl_3_) δ 14.3 (C11), 20.6 (C10), 23.3 (C4), 25.5 (C7), 32.9 (C3), 66.8 (C1), 71.8 (C2), 127.1, 129.1 and 132.0 (C5H, C6H, C8H and C9H); HRMS–ESI [M + H]^+^ calcd for C_11_H_21_O_2_, 185.1542; found, 185.1539. Enantiomeric excess was determined by HPLC analysis (Chiralcel OD-H, hexane/2-propanol 95:5, 1.0 mL min^−1^): *t*_R_ 14.7 min (minor), 15.4 (major).
